# Pioglitazone Ameliorates Atorvastatin-Induced Islet Cell Dysfunction through Activation of FFA1 in INS-1 Cells

**DOI:** 10.1155/2019/5245063

**Published:** 2019-02-03

**Authors:** Kongbo Zhu, Linglin Qian, Yanshan Lin, Li An, Guangjian Mu, Genshan Ma, Liqun Ren

**Affiliations:** ^1^Department of Cardiology, Zhongda Hospital, School of Medicine, Southeast University, No. 87 Dingjiaqiao Road, Nanjing 210009, China; ^2^Department of Geriatrics, Zhongda Hospital, School of Medicine, Southeast University, No. 87 Dingjiaqiao Road, Nanjing 210009, China

## Abstract

Increasing evidence shows that statins increase the risk of new-onset diabetes mellitus, but the exact mechanism is not clearly known. Free fatty acid receptor 1 (FFA1) has been recognized to mediate insulin secretion, and pioglitazone has direct effects on glucose-stimulated insulin secretion in addition to the reversion of insulin resistance. In this study, we found that atorvastatin decreased potassium-stimulated insulin secretion and inhibited the expression of FFA1, PDX-1, and BETA2/NeuroD in INS-1 cells. Further study demonstrated that pioglitazone prevented the impairment of insulin secretion induced by atorvastatin and enhanced the expression of FFA1, PDX-1, and BETA2/NeuroD reduced by atorvastatin in INS-1 cells. In addition, the preventive effect of pioglitazone on atorvastatin-induced impairment of insulin secretion and the enhancement of the expression of PDX-1 and BETA2/NeuroD was abolished by knockdown of FFA1 using siRNA or the PLC inhibitor, U-73122, respectively. Ultimately, FFA1 may mediate the atorvastatin-induced pancreatic *β*-cell dysfunction and pioglitazone may ameliorate this deleterious effect through the upregulation of FFA1 expression.

## 1. Introduction

Statins are potent and specific competitive inhibitors of 3-hydroxy-3-methyl-glutaryl-CoA reductase (HMG-CoA reductase), which is the rate-limiting enzyme that catalyses the conversion of HMG-CoA to mevalonate in the biosynthesis of cholesterol. Statins are widely used as plasma cholesterol-lowering drugs and are efficient in the primary and secondary prevention of both atherosclerotic cardiovascular disease and stroke. However, meta-analyses of previous studies [1–3] done with statins have shown that statins can dose-dependently increase the risk of new-onset diabetes mellitus (NODM). Diabetes has become a global epidemic disease. However, the exact mechanism of statin-induced NODM is not clearly known. Further studies on the mechanism of statin-induced NODM have important clinical significance.

A recent clinical study has proved that the increased risk is associated with an impaired insulin sensitivity and insulin secretion [4]. Statins inhibit insulin synthesis and secretion by multiple mechanisms in pancreatic *β*-cells [5–7]. The free fatty acid (FFA) receptor, free fatty acid receptor 1 (FFA1), or G protein-coupled receptor 40 (GPR40) is a member of the G protein-coupled receptors highly expressed in rodent and human pancreatic *β*-cells [8, 9]. FFA1 has been recognized to mediate insulin secretion in a phospholipase C- (PLC-) dependent manner; hence, it plays an important role in type 2 diabetes mellitus [10–14]. It is well known that thiazolidinediones (TZDs), synthetic ligands for peroxisome proliferator-activated receptor-*γ* (PPAR-*γ*), cause their glucose-lowering effects principally via reversing insulin resistance. However, some studies indicate that TZDs have direct effects on glucose-stimulated insulin secretion and protect *β*-cells [15, 16]. Furthermore, it has been reported that TZDs protect *β*-cells from FFA toxicity [17, 18], endoplasmic reticulum stress [19], and the proinflammatory cytokines [20]. TZDs increase intracellular calcium mobilization and insulin secretion mediated by the upregulation of FFA1 expression in INS-1 cells [16].

From these findings, we hypothesized that FFA1 is linked to statin-induced pancreatic *β*-cell dysfunction and that TZDs may ameliorate this deleterious effect. Hence, in this context, we investigated whether pioglitazone can ameliorate insulin secretion and synthesis dysfunction induced by atorvastatin mediated by the upregulation of FFA1 expression.

## 2. Methods

### 2.1. Cell Culture

Rat pancreatic INS-1 cells were kindly provided by Prof. Hai Qian (China Pharmaceutical University, Nanjing, China) [21]. The cells were routinely reseeded every 2-3 days and cultured in RPMI 1640 medium containing 11 mmol/L (mM) glucose, 2 mM L-glutamine supplemented with 10% heat-inactivated fetal bovine serum (FBS), 1 mM sodium pyruvate, 50 *μ*mol/L (*μ*Μ) *β*-mercaptoethanol, 10 mM N-2-hydroxyethyl piperazine-N-2-ethane-sulphonic acid (HEPES), 100 IU/mL penicillin, and 100 mg/mL streptomycin in a humidified cell incubator at 37°C in a humidified atmosphere (5% CO_2_ and 95% air). Lek Pharmaceuticals d.d. generously provided us with atorvastatin (Ljubljana, Slovenija). Final concentrations of atorvastatin ranged from 0.2 *μ*M to 20 *μ*M. Pioglitazone hydrochloride, U-73122, and GW1100 were purchased from MedChem Express (NJ, USA). INS-1 cells were incubated with atorvastatin, pioglitazone, GW1100, FFA1 siRNA, and/or U-73122 for 24 h prior to insulin secretion and other experiments. Atorvastatin, pioglitazone, GW1100, and U-73122 were dissolved in dimethyl sulfoxide (DMSO), and the final concentration of DMSO was adjusted to 0.1% (*v*/*v*). The medium containing the same amount of DMSO was used as the control.

### 2.2. Insulin Secretion Assay

INS-1 cells were seeded in 24-well plates for potassium-stimulated insulin secretion (KSIS) assays. After 24 h of incubation with medicine, the medium was removed and cells were washed once with HEPES-balanced Krebs-Ringer bicarbonate buffer (119 mM NaCl, 4.74 mM KC1, 2.54 mM CaCl_2_, 1.19 mM MgCl_2_, 1.19 mM KH_2_PO_4_, 25 mM NaHCO_3_, and 10 mM HEPES containing 0.5% bovine serum albumin (BSA), at pH 7.4) with 2.8 mM glucose. Next, cells were preincubated for 0.5 h in KRB buffer with 2.8 mM glucose. After washing twice with KRB buffer, INS-1 cells were incubated for 2 h in KRB buffer with 2.8 mM glucose or 50 mM KCl. When 50 mM K^+^ was used, Na^+^ was equally reduced to keep the osmolarity. The media were then collected and assayed for insulin levels using a rat ELISA Kit (Joyee Biotechnics Co. Ltd., Anhui, China). Insulin secretion data were normalized to total protein content in the same well. Total protein was extracted using a whole-cell lysis assay (Nanjing Keygen Biotech Co. Ltd., Nanjing, China). Total protein concentration was determined with a Bicinchoninic Acid Assay (Beyotime Biotechnology, Shanghai, China).

### 2.3. Knockdown of FFA1 with siRNA

We used the siRNA sequences targeting FFA1 (target sequence of 5′-GCTTGGTCTACACTCTCCA-3′, corresponding to position 126–144 of rat FFA1 mRNA) [22]. The negative control siRNA (NC-siRNA) was used for cell transfection as a negative control to rule out any nonspecific effects of the siRNA transfection. Transfection of siRNA was accomplished with Lipofectamine 2000 (Invitrogen, Carlsbad, CA). After 6 h, the transfection solution was replaced with medium containing 10% FBS. The efficiency of FFA1 knockdown was confirmed by quantitative real-time PCR.

### 2.4. Quantitative Real-Time PCR (qRT-PCR)

Total RNA was extracted from the cells using the TRIzol Reagent (Invitrogen, CA, USA). cDNA was transcribed from 1.5 *μ*g of RNA using a high-capacity cDNA archive kit (Applied Biosystems, CA, USA) following the manufacturer's instructions. Real-time PCR was performed with a ViiA 7 Real-Time PCR System (Applied Biosystems) using SYBR Green Real-Time PCR Master Mix (Arraystar Inc.). Real-time PCR was performed as follows: 40 cycles of PCR (95°C for 10 s, 60°C for 1 min) after initial denaturation for 10 min at 95°C. Primers used for real-time PCR are as follows: rat FFA1 5′-CCCTTGGTTATCACTGCTTTCTG-3′ (forward) and 5′-GAGCCTTCTAAGTCCGGGTTTAT-3′ (reverse), insulin 5′-ACCCAAGTCCCGTCGTGAAGT-3′ (forward) and 5′-ATCCACAATGCCACGCTTCTG-3′ (reverse), BETA2/NeuroD 5′-ATCAATCTTCTCCTCGGGTGC-3′ (forward) and 5′-GAATGGTGAAACTGACGTGCC-3′ (reverse), PDX-1 5′-AAAAGCCAGTGGGCAGGAGG-3′ (forward) and 5′-TTCCACTTCATGCGACGGTTT-3′(reverse), and *β*-actin 5′-CCTGTACGCCAACACAGTGC-3′ (forward) and 5′-ATACTCCTGCTTGCTGATCC-3′ (reverse). Expressions of FFA1, insulin, PDX-1, and BETA2/NeuroD were normalized to that of *β*-actin.

### 2.5. Western Blot Assay

Total protein and membrane protein from cells was extracted using a whole cell lysis assay (Nanjing Keygen Biotech Co. Ltd., Nanjing, China) and a Membrane and Cytosol Protein Extraction Kit (Beyotime Biotechnology, Shanghai, China), respectively. Protein concentrations were determined using the Bicinchoninic Acid Assay (Beyotime Biotechnology, Shanghai, China). The proteins were separated by 8-10% SDS-PAGE electrophoresis and transferred to nitrocellulose membranes (Millipore, Billerica, MA). The membranes were blocked for 1 h in Tris-buffered saline and Tween 20 (TBST, pH 7.6) containing 5% nonfat milk powder at room temperature and probed at 4°C overnight with primary antibodies for FFA1 (1 : 500 dilution, Santa Cruz Biotechnology, USA), PDX-1 (1 : 1000 dilution, Abcam, USA), BETA2/NeuroD (1 : 500 dilution, Proteintech, USA), and *β*-actin (1 : 1000 dilution, Proteintech, USA). The membranes were then washed three times with TBST for 15 min each and incubated with anti-rabbit secondary antibodies (1 : 5000 in TBST) conjugated to horseradish peroxidase for 1 h at room temperature. The blots were then developed in the dark by using the ECL detection kit (Proteintech). Band intensities were quantified by ImageJ 1.45 software (NIH, USA) and normalized with *β*-actin as the internal control.

### 2.6. Statistical Analysis

Data are presented as the means ± standard deviations. Statistical analysis was performed using Student's *t*-test for unpaired data when two samples were compared. Statistical significance was determined using one-way ANOVA to correct for multiple comparisons. All experiments were performed at least three times and analyzed using the statistical software SPSS for Windows (Version 17.0; SPSS). Differences with *P* < 0.05 were considered significant.

## 3. Results

### 3.1. Atorvastatin Increased Basal Insulin Secretion and Decreased Potassium-Stimulated Insulin Secretion in INS-1 Cells

**Figure 1:**
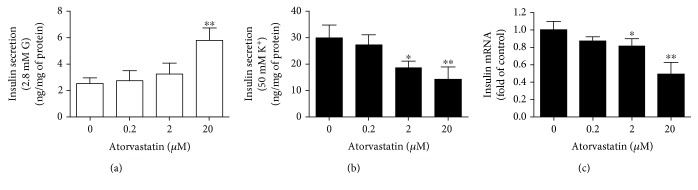
Effect of atorvastatin on basal insulin secretion and potassium-stimulated insulin secretion in INS-1 cells. (a) 20 *μ*M atorvastatin increased basal insulin secretion. (b) 2 *μ*M and 20 *μ*M atorvastatin significantly reduced the potassium-stimulated insulin secretion after 24 h of incubation. (c) qRT-PCR assay analysis of insulin expression in INS-1 cells treated with different concentrations of atorvastatin for 24 h. Values presented are the means ± standard deviations of three independent experiments. ^∗∗^*P* < 0.05 and ^∗^*P* < 0.01 compared to 0 *μ*M atorvastatin treatment.

To study the effects of atorvastatin treatment on insulin release, first we investigated the dose-response curve of atorvastatin on basal insulin secretion. As shown in Figure 1, basal insulin secretion was slightly, but not significantly, increased after incubation with 0.2 *μ*M and 2 *μ*M atorvastatin. Interestingly, 20 *μ*M atorvastatin markedly increased basal insulin secretion by 128% (Figure 1(a)). On the contrary, exposure to the higher dose of atorvastatin (2 *μ*M and 20 *μ*M) significantly reduced the potassium-stimulated insulin secretion (Figure 1(b)). Incubation with 2 *μ*M and 20 *μ*M atorvastatin reduced potassium-stimulated insulin secretion by 38% and 53%, respectively.

The effect of atorvastatin on the mRNA expression of insulin in INS-1 cells was also studied. INS-1 cells were cultured in medium containing concentrations of atorvastatin ranging from 0.2 *μ*M to 20 *μ*M for 24 hours. As shown in Figure 1(c), the mRNA expression of insulin was significantly inhibited in a dose-dependent manner in the treatment group compared to the control group.

### 3.2. Atorvastatin Inhibited the Expression of FFA1, PDX-1, and BETA2/NeuroD in INS-1 Cells

**Figure 2:**
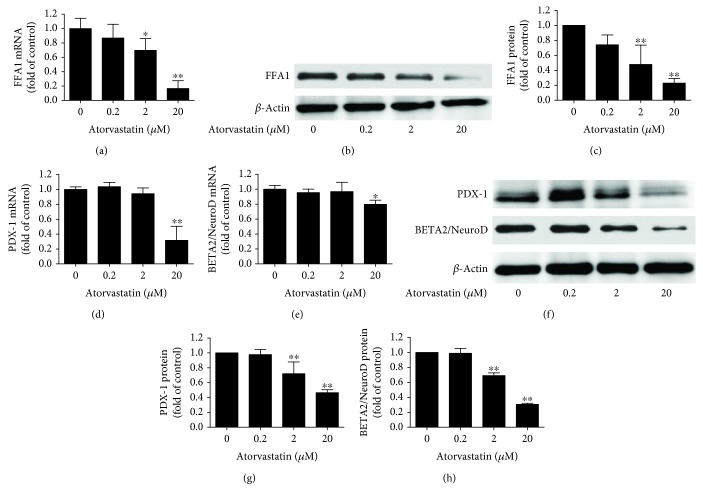
Effect of atorvastatin on the expression of FFA1, PDX-1, and NeuroD in INS-1 cells. (a) qRT-PCR assay analysis of FFA1 expression in INS-1 cells treated with different concentrations of atorvastatin for 24 h. (b and c) Western blot assay analysis of FFA1 expression in INS-1 cells treated with different concentrations of atorvastatin for 24 h. (d) qRT-PCR assay analysis of PDX-1 expression in INS-1 cells treated with different concentrations of atorvastatin for 24 h. (e) qRT-PCR assay analysis of BETA2/NeuroD expression in INS-1 cells treated with different concentrations of atorvastatin for 24 h. (f, g, and h) Western blot assay analysis of PDX-1 and BETA2/NeuroD expression in INS-1 cells treated with different concentrations of atorvastatin for 24 h. *β*-Actin was detected as control. Each experiment was repeated at least three times. ^∗^*P* < 0.05 and ^∗∗^*P* < 0.01 compared to 0 *μ*M atorvastatin treatment.

Using qRT-PCR and western blot assay, we demonstrated that treating INS-1 cells with different concentrations of atorvastatin for 24 h led to a dramatic decrease in FFA1 expression; the decrease in mRNA and protein was 83% to 77% with 20 *μ*M atorvastatin incubation for 24 h (Figures 2(a)–2(c)). To gain an insight into the *β*-cell functional adaptation, we further examined the roles of atorvastatin inhibition on specific expression of the *β*-cell transcription factors PDX-1 and BETA2/NeuroD. After treatment with 20 *μ*M atorvastatin incubation for 24 h, the level of expression of the *β*-cell specific genes, including PDX-1 and BETA2/NeuroD, decreased significantly (Figures 2(d)–2(h)).

### 3.3. Pioglitazone Prevented the Impairment of Insulin Secretion Induced by Atorvastatin in INS-1 Cells

**Figure 3:**
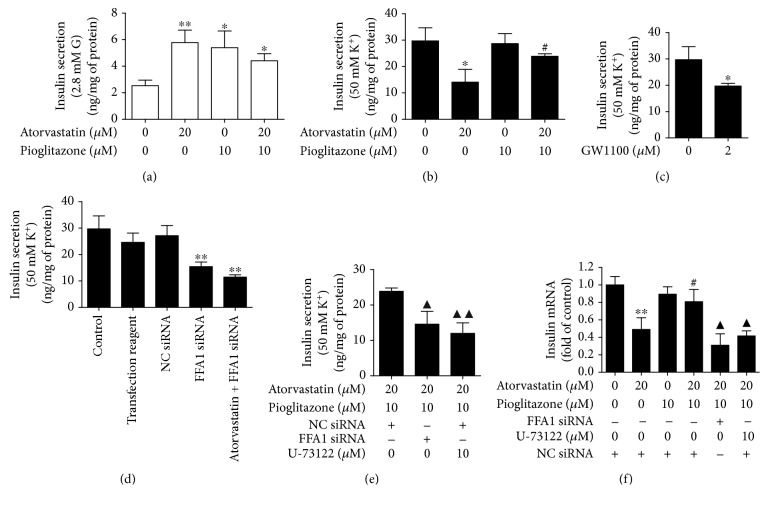
Effect of atorvastatin, pioglitazone, and FFA1-PLC signaling pathway inhibitors on basal insulin secretion and potassium-stimulated insulin secretion in INS-1 cells. (a) Administration of 10 *μ*M pioglitazone for 24 h preserved the increased basal insulin secretion by 20 *μ*M atorvastatin. (b) Administration of 10 *μ*M pioglitazone for 24 h prevented the reduction in potassium-stimulated insulin secretion induced by 20 *μ*M atorvastatin. (c) FFA1 antagonist 2 *μ*M GW1100 significantly reduced the potassium-stimulated insulin secretion after 24 h of incubation. (d) Knockdown of FFA1 using siRNA significantly reduced the potassium-stimulated insulin secretion. Atorvastatin and FFA1 siRNA together also decreased the potassium-stimulated insulin secretion. (e) The preventive effect of pioglitazone on the atorvastatin-induced impairment of insulin secretion was abolished by knockdown of FFA1 using siRNA or the PLC inhibitor U-73122 in INS-1 cells. (f) qRT-PCR assay analysis of insulin expression in INS-1 cells treated with atorvastatin, pioglitazone, FFA1 siRNA, and U-73122 for 24 h. *β*-Actin was detected as control. Negative control siRNA (NC-siRNA) was used for cell transfection as negative control to rule out any nonspecific effects that the siRNA transfection might have on insulin. Values presented are the means ± standard deviations of three independent experiments. ^∗^*P* < 0.05 and ^∗∗^*P* < 0.01 compared to control. ^#^*P* < 0.05 compared to 20 *μ*M atorvastatin treatment alone. ^▲^*P* < 0.05 and ^▲▲^*P* < 0.01 compared to atorvastatin and pioglitazone treatment together.

As shown in Figure 1(b), treatment with atorvastatin (2 *μ*M and 20 *μ*M) for 24 h markedly reduced KSIS in INS-1 cells, respectively. Moreover, administration of pioglitazone for 24 h at the concentration of 10 *μ*M significantly prevented the reduction in KSIS induced by 20 *μ*M atorvastatin (*P* < 0.05) (Figure 3(b)). In addition, administration of 10 *μ*M pioglitazone enhances the mRNA expression of insulin reduced by atorvastatin in INS-1 cells (*P* < 0.05) (Figure 3(f)).

### 3.4. Pioglitazone Enhanced the Expression of FFA1, PDX-1, and BETA2/NeuroD Reduced by Atorvastatin in INS-1 Cells

**Figure 4:**
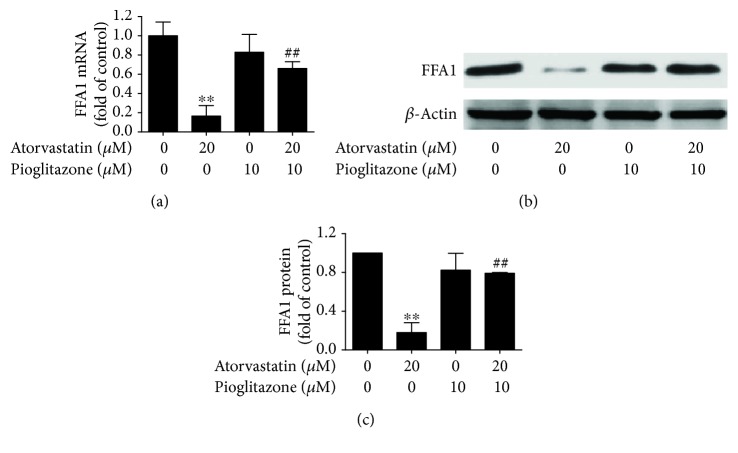
Pioglitazone increased FFA1 expression reduced by atorvastatin in INS-1 cells. (a) qRT-PCR assay analysis of FFA1 expression in INS-1 cells treated with atorvastatin and pioglitazone for 24 h. (b and c) Western blot assay analysis of FFA1 expression in INS-1 cells treated with atorvastatin and/or pioglitazone for 24 h. *β*-Actin was detected as control. Each experiment was repeated at least three times. ^∗∗^*P* < 0.01 compared to 0 *μ*M atorvastatin and 0 *μ*M pioglitazone treatment. ^##^*P* < 0.01 compared to 20 *μ*M atorvastatin treatment alone.

**Figure 5:**
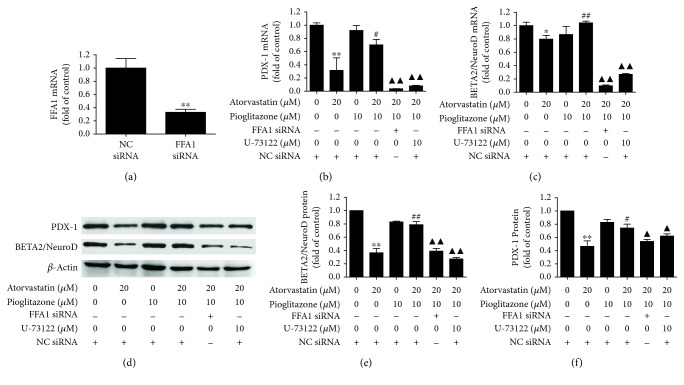
Effect of atorvastatin, pioglitazone, and FFA1-PLC signaling pathway inhibitors on the expression of PDX-1 and BETA2/NeuroD in INS-1 cells. (a) The expression of FFA1 mRNA was inhibited in rat FFA1 siRNA-transfected cells. (b and c) Administration of 10 *μ*M pioglitazone enhanced the mRNA expression of PDX-1 and BETA2/NeuroD reduced by 20 *μ*M atorvastatin in INS-1 cells, and the enhancement was suppressed by knockdown of FFA1 using siRNA or the PLC inhibitor U-73122 in INS-1 cells. (d, e, and f) Administration of 10 *μ*M pioglitazone enhanced the protein expression of PDX-1 and BETA2/NeuroD reduced by 20 *μ*M atorvastatin, and the enhancement was abolished by FFA1 siRNA and U-73122 in INS-1 cells. Negative control siRNA (NC-siRNA) was used for cell transfection as negative control to rule out any nonspecific effects that the siRNA transfection might have on cell function. *β*-Actin was detected as control. Each experiment was repeated at least three times. ^∗^*P* < 0.05 and ^∗∗^*P* < 0.01 compared to negative control. ^#^*P* < 0.05 and ^##^*P* < 0.01 compared to 20 *μ*M atorvastatin treatment alone. ^▲^*P* < 0.05 and ^▲▲^*P* < 0.01 compared to 20 *μ*M atorvastatin and 10 *μ*M pioglitazone treatment together.

In this study, atorvastatin exposure to INS-1 cells for 24 h decreased the mRNA and protein expression of FFA1 (*P* < 0.05) (Figures 2(a)–2(c)) as compared to the control in a dose-dependent manner, implying that atorvastatin impaired insulin secretion involving FFA1 and the subsequent cascade reaction in INS-1 cells. Administration of 10 *μ*M pioglitazone inhibited the reduction of FFA1 mRNA expression (*P* < 0.01) (Figure 4(a)) and protein expression (*P* < 0.01) (Figures 4(b) and 4(c)). Furthermore, administration of 10 *μ*M pioglitazone enhances the mRNA and protein expression of PDX-1 (*P* < 0.05) (Figures 5(b), 5(d) and 5(f)) and BETA2/NeuroD (*P* < 0.01) (Figures 5(c)–5(e)) reduced by 20 *μ*M atorvastatin in INS-1 cells.

### 3.5. Preventive Effect of Pioglitazone on Atorvastatin-Induced Impairment of Insulin Secretion Was Abolished by Inhibitors of FFA1-PLC Signaling Pathway in INS-1 Cells

To determine whether pioglitazone has a preventive effect on changes of insulin secretion and whether PDX-1 and BETA2/NeuroD in INS-1 cells were associated with FFA1, knockdown of FFA1 using siRNA or the PLC inhibitor U-73122 was administrated.A decrease of 67% in FFA1 mRNA expression was achieved after the siRNA transfection (Figure 5(a)). FFA1 siRNA significantly reduced the potassium-stimulated insulin secretion after 24 h of incubation (*P* < 0.01) (Figure 3(d)). Interestingly, 2 *μ*M GW1100 as a FFA1 antagonist also significantly decreased the potassium-stimulated insulin secretion after 24 h of incubation (*P* < 0.05) (Figure 3(c)). Atorvastatin and FFA1 siRNA together also decreased the potassium-stimulated insulin secretion after 24 h of incubation (*P* < 0.01) (Figure 3(d)). Notably, the improvement of KSIS by pioglitazone was blocked by FFA1 siRNA (*P* < 0.05) or 10 *μ*M U-73122 (*P* < 0.01), respectively (Figure 3(e)). Moreover, the mRNA expression of insulin enhanced by pioglitazone was abolished by FFA1 siRNA and U-73122 in INS-1 cells (*P* < 0.05) (Figure 3(f)). Additionally, the enhancement of mRNA and the protein expression of PDX-1 (*P* < 0.05) (Figures 5(b), 5(d) and 5(f)) and BETA2/NeuroD (Figures 5(c)–5(e)) was suppressed by the FFA1 siRNA or PLC inhibitor.

## 4. Discussion

Statins are widely prescribed to prevent cardiovascular disease. In recent years, it has been recognized that statins can dose-dependently increase the risk of NODM. Insulin secretion dysfunction of pancreatic beta cells is one of the most important mechanisms in the pathogenesis of type 2 diabetes. In this study, we focused on atorvastatin since it has been indicated that atorvastatin is one of the more diabetogenic statins. Here, we provide the first evidence that pioglitazone protects pancreatic *β*-cells from atorvastatin toxicity. FFA1 is linked to statin-induced pancreatic *β*-cell dysfunction, and pioglitazone increases FFA1 expression reduced by atorvastatin. As predicted, pioglitazone-induced increased insulin secretion is mediated by FFA1 induction and is blocked by the knockdown of FFA1 using siRNA or the PLC inhibitor, U-73122. In addition, the expression of PDX-1 and BETA2/NeuroD following pioglitazone treatment was upregulated in a FFA1-PLC pathway-dependent manner. Collectively, these data indicate that pioglitazone can restore insulin secretion and synthesis dysfunction induced by atorvastatin through the activation of the FFA1-PLC pathway.

Our study revealed that atorvastatin increased basal insulin secretion. Similar effects of other statins have been found before [23, 24]. The mechanism is unknown, and it is due to the unidentified off-target effects of the drug. Atorvastatin significantly reduced insulin secretion under the condition of a high (50 mM) concentration of extracellular K^+^ in a dose-dependent manner, which was compatible with previous reports [24]. However, there was no decreased glucose-induced insulin secretion in another study [24]. Differences may exist between the statins. Moreover, different concentrations and different choices of cell lines may also play a role. Pioglitazone causes a glucose-lowering effect principally via reversing insulin resistance. So far, considerable controversy exists as to whether PPAR-*γ* activation can stimulate insulin secretion in pancreatic *β*-cells [15, 16, 25–27]. It is well known that the stimulation of the FFA1 signal activates PLC resulting in the production of inositol 1,4,5-trisphosphate (IP3) and diacylglycerol (DAG). Increased IP3 binds to the IP3 receptor of the endoplasmic reticulum (ER) and mobilizes Ca^2+^ to increase intracellular Ca^2+^ concentration ((Ca^2+^)_i_) from the ER. DAG promotes F-actin remodeling and potentiates glucose-stimulated insulin secretion via protein kinase D1 [22, 28]. Here, we provide the first evidence that pioglitazone protects pancreatic *β*-cells from atorvastatin toxicity. The results in the present study showed that pioglitazone enhanced insulin secretion in INS-1 cells treated with atorvastatin for 24 h, but not in cells treated with atorvastatin and FFA1 siRNA/U-73122. This favors the view that the deleterious action of atorvastatin on the INS-1 cells is counteracted by pioglitazone through the FFA1-PLC pathway.

In this study, elevated atorvastatin exposure for 24 h led to a significant reduction in FFA1 mRNA and protein expression in INS-1 cells. In fact, FFA1 expression is decreased in type 2 diabetic islets [29]. FFA1 is a cell surface receptor expressed preferentially in pancreatic *β*-cells and in insulin-secreting *β*-cell lines. Activated FFA1 by acute exposure to FFA or agonist amplifies glucose-induced insulin secretion from pancreatic *β*-cells [13]. Therefore, FFA1 is a potential therapeutic target for the development of antidiabetic drugs. In the current study, the administration of pioglitazone inhibited the reduction of FFA1 mRNA and protein expression in INS-1 cells incubated with atorvastatin. Recent studies have also supported our results that PPAR-*γ* activation can upregulate FFA1 expression in pancreatic *β*-cells [16, 30]. Furthermore, this study demonstrated for the first time that elevated atorvastatin exposure reduced the expression of PDX-1 and BETA2/NeuroD in INS-1 cells. PDX-1 and BETA2/NeuroD are *β*-cell transcription factors that bind to different specific regions of the insulin gene and synergistically regulate insulin gene expression. It was reported that the PPAR-*γ* agonist increased the expression of PDX-1 and BETA2/NeuroD [15, 31]. Therefore, this study further investigated the effect of pioglitazone on the expression of PDX-1 and BETA2/NeuroD in INS-1 cells treated with atorvastatin. Our results showed that pioglitazone increased their expression suppressed by atorvastatin. Moreover, the enhancement of PDX-1 and NeuroD expression was inhibited by the FFA1 siRNA or PLC inhibitor. Thus, the expression of PDX-1 and BETA2/NeuroD following pioglitazone treatment was upregulated in a FFA1-PLC-dependent manner. The results imply that pioglitazone prevents the atorvastatin-induced impairment of insulin secretion and synthesis involving the FFA1-PLC signaling pathway in INS-1 cells.

In this study, FFA1-PLC signaling pathway inhibitors decreased the expression of PDX-1 and BETA2/NeuroD. These findings indicate the role of FFA1 in the atorvastatin stimulation of PDX-1 and BETA2/NeuroD expression and insulin secretion. Similar effects of FFA1 have been found before in the lipotoxicity of the pancreatic *β*-cells [17]. Nevertheless, PDX-1 can bind to an enhancer element within the 5′-flanking region of FFA1 [32] and loss of Ipf1/PDX-1 in *β*-cells impairs FFA1 expression. Therefore, further research is necessary to investigate the relationship between FFA1 and atorvastatin-affected PDX-1 and BETA2/NeuroD expression. In addition, it may be possible that the increased gene expression is due to FoxO1 nuclear exclusion by PPAR-*γ* activation [16]. However, TZDs have been identified as partial agonists at the endogenously expressed FFA1 [9, 33]. The results in the present study showed that pioglitazone enhanced insulin secretion in cells treated with atorvastatin for 24 h, but not in cells treated with the FFA1 siRNA or PLC inhibitor. Therefore, the deleterious action of atorvastatin on the *β*-cells is counteracted by pioglitazone partly through FFA1. Additional studies are required to verify the relationship between FFA1 and pioglitazone.

## 5. Conclusions

In summary, these observations suggest that FFA1 may mediate the atorvastatin-induced pancreatic *β*-cell dysfunction and pioglitazone may ameliorate this deleterious effect. Pioglitazone may restore insulin secretion and synthesis dysfunction induced by atorvastatin through the upregulation of FFA1 expression.

## Data Availability

No data were used to support this study.
